# Single lung transplantation for pulmonary fibrosis: Does side matter?

**DOI:** 10.1016/j.jhlto.2025.100229

**Published:** 2025-02-14

**Authors:** Frank Langer, Ina Starniske, Bettina Weingard, Parviz Aliyev, Migdat Mustafi, Robert Bals, Heinrike Wilkens

**Affiliations:** a Department of Thoracic Surgery, Saarland University Medical Center, Homburg, Germany; b Department of Internal Medicine V, Pneumology and Intensive Care Medicine, Saarland University Medical Center, Homburg, Germany; c Helmholtz Institute for Pharmaceutical Research Saarland (HIPS), Helmholtz Center for Infection Research (HZI), Saarland University Campus, Saarbrücken, Germany

**Keywords:** lung transplantation, single lung transplantation, interstitial lung disease, pulmonary fibrosis, donor lung sizing

## Abstract

**Background:**

The implementation of the Lung Allocation Score in the Eurotransplant international collaborative framework decreased waiting list mortality, but organ shortage remains a significant problem. Single lung transplantation (sLTx)—whenever possible—may decrease waiting list mortality. We have consistently employed sLTx for recipients with pulmonary fibrosis. In the current investigation, we sought to analyze if this strategy can lead to an acceptable long-term outcome and if the side of sLTx has an impact on the outcome.

**Methods:**

Between 1995 and 2024, we performed 138 sLTx for patients with pulmonary fibrosis (54 ± 9 years, 88 male). Data and outcomes were analyzed retrospectively comparing recipients receiving left sLTX (*n* = 98) and right sLTx (*n* = 40).

**Results:**

Survival was 83%, 59%, and 29% at 1, 5, and 10 years for the total patient cohort. Survival was similar for left and right sLTx (83 vs 81%, 58 vs 64%, and 29 vs 28% at 1, 5, and 10 years, *p* = 0.54). Left and right transplantations lead to similar best post-transplant forced expiratory volume per second (74% ± 20% vs 74% ± 21%, *p* = 0.86). While the total lung capacity (TLC) ratio TLC_donor_/predicted TLC_recipient_ was similar between groups (104% vs 100%), the ratio TLC_donor_/actual TLC_recipient_ was higher in left sLTx (185% vs 158%, *p* = 0.04). On multivariate regression analysis, postoperative pneumonia (*p* = 0.003, hazard ratio 3.404) and sepsis (*p* = 0.002, hazard ratio 10.700) were identified as predictors for early mortality.

**Conclusions:**

Performing sLTx for pulmonary fibrosis patients can be an effective strategy to optimize donor utilization and improve outcomes—irrespective of graft side.

## Background

The first lung transplantation was performed by Hardy in 1963 and the first clinically successful lung transplantation in 1983 by Cooper—both procedures were single lung transplantations (sLTx). Double lung transplantation (dLTx) was introduced later in 1988 by Patterson.^1^ DLTx was designed to overcome clinical problems, which cannot be solved by sLTx. Clear indications for dLTx are elimination of infectious load in cystic fibrosis, reduction of pulmonary vascular resistance in pulmonary arterial hypertension, or overinflation in chronic obstructive pulmonary disease/emphysema. However, sLTX may be a good option for interstitial lung disease (ILD). Pulmonary fibrosis as a restrictive pulmonary disease is frequently associated with secondary pulmonary hypertension. SLTx for pulmonary fibrosis will therefore lead to preferred ventilation of the graft and preferred perfusion of the graft resulting in an optimal ventilation/perfusion match. Moreover, sLTx accommodates a donor lung with a total lung capacity (TLC) that approximates at least the predicted TLC of the recipient, while sizing remains a difficult issue in dLTX for pulmonary fibrosis.^2^ Within the last decades, however, many centers have literally abandoned sLTx or used it only occasionally as lower-risk procedure for elderly patients with significant comorbidities. Randomized trials could provide answers to this clinical dilemma, but have not been performed, yet.

Since the Lung Allocation Score (LAS) was introduced in Germany in 2011, a decreased number of deaths among patients on the waiting list has been documented.^3^ Nevertheless death on the waiting list remains a clinical challenge due to severe organ shortage since only 20% to 30% of donor lungs are judged suitable for transplantation.^4^ Extended donor criteria have been suggested to overcome this dilemma and have become a clinical reality in daily practice.^5^ The clinical value of ex-vivo-lung perfusion for marginal donors remains to be defined, yet.^6^ Donation after circulatory death can increase the donor pool, but is currently not an accepted option in all countries. Thus, a simple method, that is, stringent sLTx for pulmonary fibrosis instead of dLTx, may help to decrease mortality on the waiting list.

Recently, a large retrospective trial employing propensity score analysis (*n* = 466 in each group) documented similar survival after sLTx and dLTx for idiopathic pulmonary fibrosis.^7^ Smits et al from Eurotransplant analyzed the outcome of 90 lung twin pairs (2 sLTx from 1 donor) operated on 16 European centers and observed more fatal complications in recipients receiving a left-sided sLTx (1-year survival: right sLTx 92%/left sLTx 62%, *p* = 0.04).^8^ We have consistently performed sLTx for patients with pulmonary fibrosis since 1995. In the current investigation, we sought to analyze if outcome and functional capacity differ in recipients undergoing left sLTx or right sLTx.

## Methods

Between October 1995 and October 2024, a total of 423 lung transplantation were performed at our center. All sLTx (*n* = 138) for ILD were included in the current retrospective investigation. To better characterize urgency for patients with ILD in the pre-LAS era, we made the following assumption: by default, we equated HU (high urgency) to a LAS of 75, U (urgent) to a LAS of 55, and T (transplantable) to a LAS of 35. Follow-up was conducted by our transplant outpatient clinics. Best forced expiratory volume per second (FEV_1_) was defined as the mean of the 2 best FEV_1_ measurements taken at least 3 weeks apart after lung transplantation. Data collection for this retrospective study was approved by the Saarland University Medical Center Transplantation Ethics Committee before initiation. All patients had signed informed consent for data collection and analysis before being admitted to the transplant waiting list. The study was conducted in compliance with the Declaration of Helsinki.

### Statistical analysis

Data were expressed as mean ± standard deviation unless otherwise specified. Statistical analysis was performed using standard software (SigmaStat, Systat). Normal distribution was assessed using the Kolmogorov-Smirnov test. Comparisons were performed between groups (normally distributed continuous data: *t*-test or an analysis of variance, non-normally distributed continuous data: Mann-Whitney-U-rank-test or analysis of variance on ranks, discrete data: Fisher's exact test or chi-square test). Kaplan-Meier analyses of survival were also calculated using standard software (Prism, GraphPad)—the log-rank test was used to compare the survival distributions. A Cox regression analysis was performed using standard software (SPSS, IBM) to identify risk factors for death after sLTx. For early mortality (i.e., survival < 1 year), recipient age, recipient sex, donor age, donor smoking status, LAS, preoperative extracorporeal membrane oxygenation (ECMO), use of extracorporeal circulation (ECC), re-exploration for bleeding, transfusion, mechanical ventilation time, intensive care unit (ICU) stay, hospital stay, postoperative pneumonia, sepsis, and acute renal failure were used as covariates.

For intermediate mortality (survival 1-10 years), recipient age, recipient sex, donor age, donor smoking status, preoperative ECMO, respiratory infections, rejections, and chronic lung allograft dysfunction (CLAD) were used as covariates.

For long-term mortality (>10 years), recipient age, recipient sex, donor age, donor smoking status, respiratory infections, rejections, and CLAD were used as covariates.

All covariates were initially assessed in univariate analyses and the significant parameters were subsequently included in multivariate analyses. The risk was expressed as hazard ratio (HR).

Statistical significance was assessed at a significance level of 5% (*p* = 0.05).

## Results

Underlying ILD was classified as idiopathic pulmonary fibrosis (*n* = 111), hypersensitive pneumonitis (*n* = 11), unspecified ILD (*n* = 5), combined pulmonary emphysema and fibrosis (predominant fibrosis *n* = 1), and acute interstitial pneumonitis (*n* = 1). Six patients with prior sLTx and bronchiolitis obliterans syndrome (BOS) underwent sLTx of the native contralateral lung and 3 patients with BOS underwent redo sLTx of the previously transplanted lung. The majority of patients were male (*n* = 88)—recipient age ranged from 24 to 68 years (mean 54 ± 9 years). The median LAS was 47.

All donor's lungs were cadaveric organs after brain death from donors within the Eurotransplant cooperation. Donor age ranged from 16 to 78 years (mean 46 ± 15 years) and donor TLC was 7.0 ± 1.0 liter.

**Figure 1:**
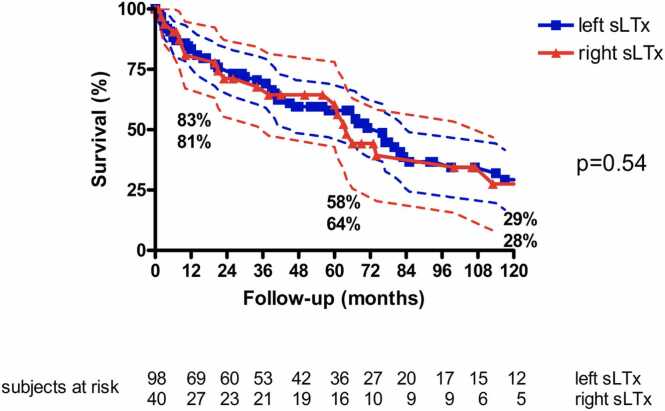
Kaplan-Meyer survival analysis: left sLTx vs right sLTx (dotted lines represent 95% confidence intervals). sLTx, single lung transplantation.

Post-transplant survival was 83%, 59%, and 29% at 1, 5, and 10 years for the total patient cohort (Figure 1). TLC (pre: 3.2 ± 1.1 liter, post: 4.6 ± 1.0; *p* < 0.001), vital capacity (VC, pre: 1.6 ± 0.7 liter, post: 2.8 ± 0.9 liter; *p* < 0.001), and FEV_1_ (pre: 1.3 ± 0.9 liter, post: 2.2 ± 1.7 liter; *p* < 0.001) increased after transplantation. The best post-transplant FEV_1_ value was 74% ± 20%.

Our current study included recipients over 3 decades. We performed a survival analysis comparing these 3 decades (1995-2004, 2005-2014, and 2015-2024) and observed no difference (*p* = 0.65) in the log-rank test.

### Left sLTx

The majority of patients underwent left sLTx (*n* = 98, 71%). The recipients were 55 ± 8 years old, and a total of 61 patients were male. The median LAS was 46.5. Donor age was 46 ± 15 years and donor TLC was 6.6 ± 1.1 liter.

Survival rates for left sLTx were 83%, 58%, and 29% at 1, 5, and 10 years, respectively. TLC (pre: 3.1 ± 1.2 liter, post: 4.5 ± 1.0; *p* < 0.001), VC (pre: 1.6 ± 0.7 liter, post: 2.8 ± 0.9 liter; *p* < 0.001), and FEV_1_ (pre: 1.4 ± 0.6 liter, post: 2.3 ± 0.7 liter; *p* < 0.001) increased after transplantation. The best post-transplant FEV_1_ value was 74% ± 20%.

### Right sLTx

Forty patients underwent right sLTx. The recipients were 54 ± 10 years old and 27 were male. The median LAS was 48. Donor age was 48 ± 13 years and donor TLC was 6.6 ± 1.2 liter.

Survival rates for right sLTx were 81%, 64%, and 28% at 1, 5, and 10 years, respectively. TLC (pre: 3.2 ± 1.0 liter, post: 4.7 ± 1.0; *p* < 0.001), VC (pre: 1.6 ± 0.6 liter, post: 3.0 ± 0.8 liter; *p* < 0.001), and FEV_1_ (pre: 1.3 ± 0.5 liter, post: 2.3 ± 0.7 liter; *p* < 0.001) increased after transplantation. The best post-transplant FEV_1_ value was 74% ± 21%.

### Comparison left sLTx vs right sLTx

There was no difference in demographic and perioperative data except for the higher need for ECC in right sLTx (55% vs 32%; *p* = 0.01; Table 1).

**Table 1:** Demographic Data of Recipients and Donors; Peri- and Postoperative Data of Recipients

Parameter	Left sLTx (*n* = 98)	Right sLTx (*n* = 40)	*p*
Recipient age (years)	55 ± 8	54 ± 10	U: 0.82
Recipient gender (male/female)	61/37	27/13	F: 0.70
Recipient height (cm)	171 ± 9	172 ± 9	*t*: 0.42
Recipient predicted TLC (liter)	6.4 ± 0.9	6.6 ± 0.8	*t*: 0.26
LAS (median)	46.5	48	U: 0.34
Donor age (years)	46 ± 15	48 ± 13	U: 0.53
Donor gender (male/female)	62/36	25/15	F: 1.0
Donor height (cm)	176 ± 9	176 ± 10	U: 0.98
Donor-predicted TLC (liter)	6.6 ± 1.1	6.6 ± 1.2	U: 0.86
Preoperative ECMO	12 (12%)	8 (20%)	F: 0.29
Intraoperative ECC	31 (32%)	22 (55%)	F: 0.01
Erythrocyte transfusion (*n*)	0.9 ± 2.4	1.6 ± 3.5	U: 0.41
Re-exploration (*n*)	15 (15%)	5 (13%)	F: 0.79
Ventilation hours (median)	34	29	U: 0.57
ICU days (median)	8	6	U: 0.05
Days in hospital (median)	27	22.5	U: 0.25
Postoperative pneumonia	25 (26%)	11 (28%)	F: 0.83
Postoperative sepsis	12 (12%)	5 (13%)	F: 1.0
Postoperative acute renal failure	24 (24%)	9 (23%)	F: 1.0
Postoperative stroke	0 (0%)	0 (0%)	F: 1.0
Rejections (median)	1	1	U: 0.72
CLAD (median)	1	0.5	U: 0.62
Hospitalizations for pneumonia (median)	1	1	U: 0.28
Survival (median)	39.5	36	U: 0.67

Abbreviations: CLAD, chronic lung allograft dysfunction; ECC, extracorporeal circulation; ECMO, extracorporeal membrane oxygenation; ICU, intensive care unit; LAS, lung allocation score; sLTx, single lung transplantation; TLC, total lung capacity.

Survival was similar for left sLTx and right sLTx (83% vs 81%, 58% vs 64%, and 29% vs 28% at 1, 5, and 10 years; *p* = 0.54, Figure 1). Left and right transplantations led to similar best post-transplant FEV_1_ values (74% ± 20% vs 74% ± 21%; *p* = 0.86, Table 2). While the TLC ratio TLC_donor_/predicted TLC_recipient_ was similar between groups (104% vs 100%), the ratio TLC_donor_/actual TLC_recipient_ was higher in left sLTx (185% vs 158%; *p* = 0.04).

**Table 2:** Functional Data Following sLTx

Parameter	Left sLTx (*n* = 98)	Right sLTx (*n* = 40)	*p*
VC preop (liter)	1.6 ± 0.7	1.5 ± 0.6	U: 0.683
VC postop (liter)	2.8 ± 0.9	3.0 ± 0.9	*t*: 0.221
FEV1 preop (liter)	1.4 ± 0.6	1.2 ± 0.5	U: 0.447
FEV1 postop (liter)	2.3 ± 0.7	2.4 ± 0.8	U: 0.530
TLC preop (liter)	3.1 ± 1.2	3.3 ± 1.0	U: 0.042
TLC postop (liter)	4.5 ± 1.0	4.8 ± 1.1	*t*: 0.232

Abbreviations: FEV1, forced expiratory volume per second; sLTx, single lung transplantation; TLC, total lung capacity; VC, vital capacity.

Surveillance bronchoscopy identified 3 bronchial anastomotic complications (2%) following left sLTx. Two patients with anastomotic stenosis underwent bronchial sleeve resection and 1 patient with bronchial dehiscence had spontaneous healing.

### Survival subgroup analysis

Recipients were stratified in 3 cohorts (Table 3): those who survived less than 1 year, 1 to 10 years, and greater than 10 years according to Jawitz et al.^9^ Ventilation hours (invasive ventilation and noninvasive ventilation, *p* = 0.03) and ICU days (*p* = 0.09) were increased in group 1. Pneumonia (*p* = 0.02), sepsis (*p* < 0.0001), and acute renal failure (*p* = 0.005) occurred also more frequently in group 1.

**Table 3:** Demographic Data of Recipients and Donors; Peri- and Postoperative Data of Recipients Stratified by Survival/Follow-up (<1, 1-10, and >10 Years)

Parameter	Survival/follow-up <1 year (*n* = 44)	Survival/follow-up 1-10 years (*n* = 79)	Survival/follow-up >10 years (*n* = 15)	*p*
Recipient age (years)	55 ± 9	55 ± 9	52 ± 10	0.43
Recipient gender (male/female)	25/19	52/27	11/4	0.44
LAS (median)	46	46	55	0.67
Donor age (years)	48 ± 15	47 ± 15	37 ± 13	0.06
Donor gender (male/female)	23/21	53/26	11/4	0.18
Preoperative ECMO	8 (18%)	10 (13%)	2 (13%)	0.70
Intraoperative ECC	16 (36%)	31 (39%)	5 (33%)	0.89
Erythrocyte transfusion (*n*)	2.00 ± 4.1	0.6 ± 1.7	1.0 ± 1.7	0.74
Re-exploration (*n*)	10 (23%)	8 (10%)	2 (13%)	0.16
Ventilation hours (median)	48.5	29	21.5	0.03
ICU days (median)	9	6	5.5	0.09
Days in hospital (median)	21	26	26.5	0.33
Postoperative pneumonia	18 (41%)	14 (18%)	4 (27%)	0.02
Postoperative sepsis	15 (34%)	2 (3%)	0 (0%)	<0.0001
Postoperative acute renal failure	17 (39%)	16 (20%)	0 (0%)	0.005
Postoperative stroke	0 (0%)	0 (0%)	0 (0%)	1.0
Rejections (median)	0	1	1	0.003
CLAD (median)	0	1	2	0.002
Hospitalizations for pneumonia (median)	1	2	2	<0.001
Survival (median)	3	58	165	<0.001

Abbreviations: CLAD, chronic lung allograft dysfunction; ECC, extracorporeal circulation; ECMO, extracorporeal membrane oxygenation, ICU, intensive care unit, LAS, lung allocation score.

A Cox regression analysis was employed to identify predictors for death in these 3 groups. On univariate analysis, perioperative transfusion (*p* = 0.04, HR 1.151), ventilation hours (*p* = 0.002, HR 1.001), ICU stay (*p* < 0.001, HR 1.016), postoperative pneumonia (*p* < 0.001, HR 3.262), sepsis (*p* < 0.001, HR 9.845), and acute renal failure (*p* < 0.001, HR 3.276) were identified as independent risk factors for early mortality (survival <1 year, Table 4a). Donor age (*p* = 0.019, HR 1.027) was identified as an independent risk factor for intermediate mortality (survival 1-10 years, Table 4b). No significant predictor for late mortality (>10 years, Table 4c) could be identified.

**Table 4a:** Cox Regression Analysis: Risk Factors for Early Mortality (i.e., Survival < 1 Year)

Risk factors for early mortality (survival < 1 year, univariate analysis)	*p*
Recipient age	0.804
Recipient gender	0.637
LAS	0.964
Donor age	0.724
Donor smoking status (0-10 py, 11-20 py, 21-30 py)	0.656
Preoperative ECMO	0.203
Intraoperative ECC	0.748
Erythrocyte transfusion	0.040
Re-exploration	0.073
Ventilation hours	0.002
ICU days	<0.001
Days in hospital	0.230
Postoperative pneumonia	<0.001
Postoperative sepsis	<0.001
Postoperative acute renal failure	<0.001
	
*Risk factors for early mortality (survival < 1 year, multivariate analysis)*	*p*
Postoperative pneumonia	0.003
Postoperative sepsis	0.002

Abbreviations: ECC, extracorporeal circulation; ECMO, extracorporeal membrane oxygenation, ICU, intensive care unit; LAS, lung allocation score.

**Table 4b:** Cox Regression Analysis: Risk Factors for Intermediate Mortality (Survival 1-10 Years)

Risk factors for intermediate mortality (survival 1-10 years, univariate analysis)	*p*
Recipient age	0.117
Recipient gender	0.859
Preoperative ECMO	0.760
Donor age	0.019
Donor smoking status (0-10 py, 11-20 py, 21-30 py)	0.629
Hospitalizations for pneumonia	0.375
Rejections	0.230
CLAD	0.356

Abbreviations: CLAD, chronic lung allograft dysfunction; ECMO, extracorporeal membrane oxygenation.

**Table 4c:** Cox Regression Analysis: Risk Factors for Late Mortality (i.e., Survival > 10 Years)

Risk factors for late mortality (survival > 10 years, univariate analysis)	*p*
Recipient age	0.183
Recipient gender	0.756
Donor age	0.198
Donor smoking status (0-10 py, 11-20 py, 21-30 py)	0.509
Hospitalizations for pneumonia	0.405
Rejections	0.117
CLAD	0.611

Abbreviation: CLAD, chronic lung allograft dysfunction.

On multivariate analysis, postoperative pneumonia (*p* = 0.003, HR 3.404) and sepsis (*p* = 0.002, HR 10.700) were identified as predictors for early mortality (survival <1 year).

## Discussion

The LAS system was implemented in the United States in 2005 and in 2011 in Germany. With this allocation model, a decrease in mortality on the waiting list was observed in both countries.^3^, ^10^ While in Germany up to every fifth patient died on the waiting list before the advent of the LAS system, mortality was reduced since then by 25%.^3^ Nevertheless death on the waiting list remains an issue—particularly in Germany with an extremely low organ donation rate (2023: 114 organ donors per million citizens). This is a result of the still existing opting-in legislation and the lack of donation after circulatory death legislation in Germany.

Single-center studies and registry-based studies have reported periprocedural and long-term outcomes after sLTX and dLTx. However, no prospective randomized trials have ever been conducted to document the individual merit of both procedures.

Is there a difference in survival between sLTX and dLTX for patients with ILD? Meyers et al reported the first larger single-center cohort of recipients with pulmonary fibrosis and did not observe a survival difference between sLTx vs dLTx.^11^ In their series, they performed either sLTX or dLTx based primarily on organ availability. Multiple investigators employed the United Network for Organ Sharing (UNOS) Thoracic Transplant database to compare outcomes of sLTx and dLTx in patients with pulmonary fibrosis. Thabut et al observed similar survival for sLTx and dLTx in patients with pulmonary fibrosis.^12^ Primary graft failure was a more common cause of death in patients undergoing dLTx in this analysis, while cancer was a more common cause of death in recipients of a sLTx. Weiss et al analyzed short-term survival (up to 1 year) and documented a 14% decrease in mortality with dLTx in high-risk patients based on the LAS.^13^ Speicher et al observed improved 5-year survival by dLTx in patients >65 years.^14^ Schaffer et al documented better long-term survival with dLTx,^15^ while Chauhan et al did not observe a survival difference in patients who were concurrently listed for sLTx or dLTx.^16^ The most recent study by Ranganath et al employed propensity score matching to compare outcomes of sLTX and dLTx. This study demonstrated similar long-term survival (up to 10 years) in both groups (*n* = 466 in each group). Additionally, sLTx recipients were less likely to require prolonged (>48 hours) ventilator support and showed a trend toward a lower rate of post-transplant renal failure and shorter hospital stays.^7^ With this conflicting data arising from the UNOS Thoracic Transplant database, a meta-analysis was performed by Li et al, who integrated 16 studies including 17,872 patients with pulmonary fibrosis (10,215 sLTx, 7,657 dLTx). Survival rates at 1 year and 5 years following sLTx and dLTx were 78.4% vs 79.6% and 54.9% vs 51.1%, respectively. The authors concluded that there was no difference in long-term survival in patients undergoing sLTx or dLTx.^17^ Our observed survival rates of 83% at 1 year and 59% at 5 years after sLTx compare well with these international results.

Is there a difference in postoperative functional capacity between sLTX and dLTX for patients with ILD? Gerbase et al^18^ reported that sLTx recipients, on average, had 20% lower FEV_1_ values. However, the performance in the 6-minute walk test and quality of life questionnaires were similar for sLTX and dLTx recipients. Mason et al documented the improvement in lung function in over 460 recipients of sLTx and dLTx for different underlying pulmonary diseases.^19^ The values of FEV_1_ (65%, 58%, and 59% vs 51%, 43%, and 40%, *p* = 0.03) as well as VC at 1, 3, and 5 years were better after dLTx than after sLTx, but never approached double that of sLTx. Despite these findings favoring dLTx, the benefit of dLTx over sLTx appeared to be much smaller than expected—the explanation for this remains unclear. Interestingly, our sLTx patients with ILD achieved postoperative FEV_1_ values of 74%—irrespective of graft side.

Is there a difference between left and right sLTx? This question had already been addressed by Tsagkaropoulos et al^20^—however in a heterogeneous patient population. The most common underlying diseases in this study were chronic obstructive pulmonary disease/emphysema in 55.6% and ILD in 36.6%. Survival at 1 and 5 years was 78.4% and 49.4%, respectively, with no significant differences between left and right sLTx. FEV1 improved in both groups to comparable values up to 36 months. The authors concluded that the graft side did not influence survival, freedom from BOS, complications, or pulmonary function after sLTx.

Lung twinning—that is, performing 2 sLTx from 1 donor—is an ideal model to further evaluate this question. Smits et al from Eurotransplant analyzed the outcome of 90 lung twin pairs operated in 16 European centers.^8^ In her analysis, more fatal complications were observed in recipients receiving a left-sided sLTx. The outcome was particularly worse if the retrieval center was different from the transplant center (1-year survival: right sLTx 92%/left sLTx 62%, *p* = 0.04). Snell et al reported the largest single-center experience of lung twinning with 38 pairs of recipients.^21^ This Australian group did not observe different outcomes between the first and second twins. However, this group reported an inferior intermediate outcome of left-single lung recipients—primarily related to increased mortality from airway complications.^21^ In our series of lung twins,^22^ we did not observe bronchial complications in 32 paired sLTx, but in our total cohort of recipients with pulmonary fibrosis, all 3 bronchial complications were seen in the left sLTx.

**Figure 2:**
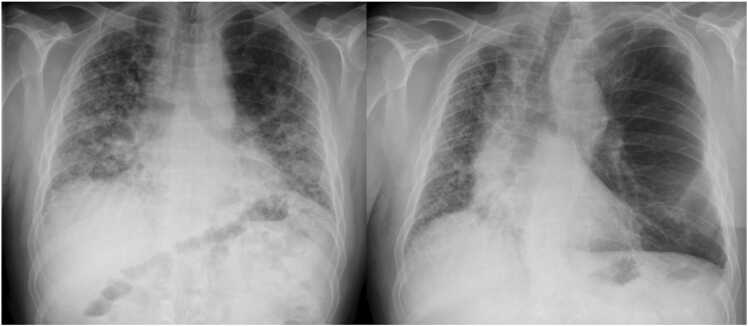
Example of a left sLTx in a 54-year-old male with pulmonary fibrosis (left: preoperative, right: postoperative). sLTx, single lung transplantation.

**Figure 3:**
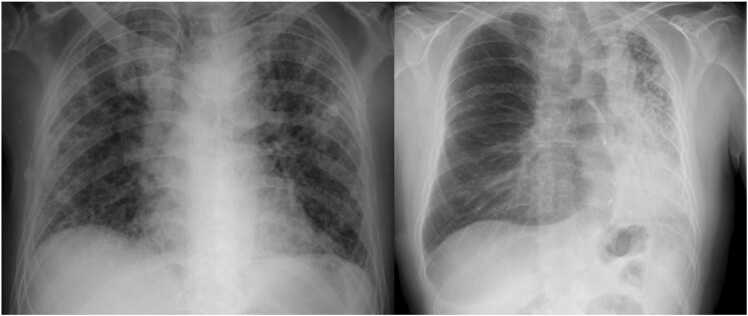
Example of a right sLTx in a 48-year-old male patient with pulmonary fibrosis as part of Hermansky-Pudlak syndrome (left: preoperative, right: postoperative). sLTx, single lung transplantation.

Is sizing different for sLTx and dLTx? The allocation of donor lungs is typically based on the blood group and the predicted TLC. The vast experience with sizing of donor lungs is provided in a review by Barnard et al.^2^ According to the ISHLT consensus report on lung donor acceptability criteria donor lungs for dLTx should be between 75% and 125% of the recipient predicted TLC. This rule works well for obstructive, suppurative, and pulmonary vascular disease, but not for ILD. Some centers therefore still use size measurements based on chest X-rays, while some employ CT volumetry.^23^ Barnard et al recommend to accept a donor TLC, that is halfway between the recipients' predicted and actual TLC values.^2^ Research is lacking evidence that characterizes sizing for sLTx in recipients with ILD such as pulmonary fibrosis.^2^ There are only 2 studies by Miyoshi et al^24^, ^25^ in very small patient cohorts (*n* = 8, *n* = 15), which both came up with similar results. The investigators concluded that in sLTx for pulmonary fibrosis, a sLTx organ into the left chest may expand to its own (donor) size, while a sLTx organ into the right chest may only expand to the recipient's normal (predicted) thoracic volume.^24^, ^25^ We were not able to support this hypothesis with similar correlations. Either because we used only pulmonary function tests instead of perfusion studies to quantify perfusion of the graft or we had the larger patient series. Based on our results (ratio TLC_donor_/predicted TLC_recipient_: left sLTx 104% vs right sLTx 100%), we would support the hypothesis that sLTx accommodates donor lungs in the range of the predicted TLC of the donor. Particularly, the left chest expands significantly due to mediastinal shift and descent of the diaphragm (ratio TLC_donor_/actual TLC_recipient_: left sLTx 185% vs right sLTx 158%, *p* = 0.04)—even though our left sLTx recipients had severe restriction (median actual TLC: left sLTx 2.8 vs right sLTx 3.4 liter; *p* = 0.042). In our series, we have performed predominantly left sLTx for 2 reasons: first, the left phrenic nerve passes further away from the hilus than on the right side. Second, we followed the observations by Miyoshi et al^24^, ^25^—but based on our current data with a much larger patient series, we can conclude that the side of sLTx does not matter. In fact, we recommend using donor lungs in the range of 100% to 120% of the predicted recipient TLC for sLTx—irrespective of side (Figures 2 and 3). We are convinced that this sizing strategy in conjunction with the optimal ventilation/perfusion match physiology in ILD recipients is the key to the observed excellent functional capacity (FEV_1_ 74%—irrespective of graft side).

What happens to the native ILD lung after sLTx? The remaining native lung might cause morbidity (increased risk of infection related to the structurally damaged lung, pneumothorax), and even mortality (lung cancer). Pneumothorax may be troublesome in patients with ILD when a noncompliant lung will not expand sufficiently. In our patient cohort with significant mediastinal shift due to true-sized or oversized allografts, we have never observed such problems. However, we did observe 6 cases of lung cancer in the native lung in our patient series with 4 resulting deaths. According to the current literature,^12^, ^17^ sLTx is associated with a higher incidence of lung cancer than dLTx, which might be attributed to the remaining native lung (ILD, diminished tumor suppression due to immunosuppression).

What happens with the remaining donor lung in case of sLTx? A recent analysis based on the UNOS database documented that in only 43% of harvests for sLTx both donor lungs were used.^14^ It appears unlikely that unilateral pathology (i.e., aspiration, contusions, lacerations, bullae, etc.) prevented the contralateral lung from being used in the remaining 57%. If the other lung is perfect, the contralateral suboptimal lung may still be used in case of dLTx. The patient will survive with 1 perfect lung and the suboptimal lung will recover in the weeks thereafter. A similar study within the Eurotransplant region is lacking. But with the severe organ shortage in Germany and the resulting trend toward the use of marginal organs, it is unlikely that a similar amount of potentially transplantable organs is wasted. In contrast, we have accepted lung twinning whenever possible. This procedure exposes the recipient of the second lung to an increased ischemic time, but it allows the ultimate use of existing donor lungs. In a previously published study, we documented the results of 16 of such procedures^22^—meanwhile, we have performed 20 twinning transplantations resulting in 40 sLTx.

**Figure 4:**
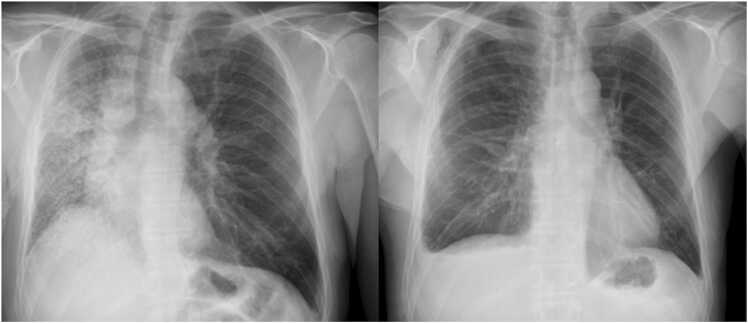
Example of a simple redo sLTx, that is, transplantation of the native right lung with pulmonary fibrosis, in a 54-year-old male with bronchiolitis obliterative syndrome after previous left sLTx (left: preop, right: postop). sLTx, single lung transplantation.

In summary, there is no survival difference after sLTx and dLTx in patients with ILD, but postoperative functional capacity is only slightly better after dLTx. Ideally, the benefits of a dLTx should justify the allocation of 2 lifesaving organs to a single patient. However even though clear evidence is lacking, 75% of lung transplantations are nowadays performed as dLTx.^26^ SLTx is frequently performed only for elderly fragile recipients with comorbidities—this may explain why sLTx was identified as an independent risk factor for long-term survival.^9^ In contrast, we have consistently performed sLTx in all patients with ILD—irrespective of age. In fact, we do believe that sLTx is a good option in younger patients with ILD, who will qualify as candidates for redo transplantation in the future. Redo transplantation will then involve the native contralateral lung (Figure 4) and is less complex than a “real” redo transplantation after dLTx. Our cohort includes 3 “real” redo sLTx, who all died acutely, as well as 6 redo sLTx of the native contralateral side (3 deaths within the first year).

### Limitations

The limitations of this study should be acknowledged. As a single-center analysis, the findings may not be fully generalizable to other institutions or patient populations. The retrospective nature of the study introduces the potential for selection bias, and there may be confounding factors that were not accounted for. Additionally, the heterogeneity of the patient cohort, including variations in disease severity, age, and comorbidities, may influence the results. While the data provides valuable insights into the outcomes of sLTx for pulmonary fibrosis, prospective multicenter studies would be necessary to validate these findings and reduce the risk of bias.

## Conclusions

We conclude that the stringent successful use of sLTX for pulmonary fibrosis—including lung twinning whenever possible—may expand the donor pool and may help to further reduce waiting list mortality. Therefore, centers should reconsider their individual donor profiles and the potential for sLTx in pulmonary fibrosis cases.

## Ethics declaration

The study was conducted in compliance with the Declaration of Helsinki. All patients had signed informed consent for data collection and analysis before admission to the transplant waiting list. Data collection for the current retrospective investigation was approved by the Saarland University Medical Center Transplantation Ethics Committee before data collection.

## Author contributions

F. Langer: conception and design of the study, data collection and analysis, writing of the manuscript. B. Weingard, P. Aliyev, M. Mustafi, R. Bals and H. Wilkens: patient care, revision of the manuscript. All authors read and approved the final manuscript for publication.

## Disclosure statement

The authors have no conflicts of interest to disclose.

The authors gratefully acknowledge the support of our lung transplant program provided by Gerd Beck, Arnstein.

This research did not receive any funding from agencies in the public, commercial or not-for-profit sectors.

## Data Availability

All clinical data can be accessed in the institutional lung transplant database.

## References

[1] Cooper J.D. (1997). The history of surgical procedures for emphysema. Ann Thorac Surg.

[2] Barnard J.B., Davies O., Curry P. (2013). Size matching in lung transplantation: an evidence-based review. J Heart Lung Transplant.

[3] Gottlieb J., Smits J., Schramm R. (2017). Lung transplantation in Germany since the introduction of the lung allocation score. Dtsch Arztebl Int.

[4] de Perrot M., Liu M., Waddell T.K., Keshavjee S. (2003). Ischemia-reperfusion-induced lung injury. Am J Respir Crit Care Med.

[5] Smits J.M., van der Bij W., Van Raemdonck D. (2011). Defining an extended criteria donor lung: an empirical approach based on the Eurotransplant experience. Transpl Int.

[6] Warnecke G., Van Raemdonck D., Smith M.A. (2018). Normothermic ex-vivo preservation with the portable Organ Care System Lung device for bilateral lung transplantation (INSPIRE): a randomised, open-label, non-inferiority, phase 3 study. Lancet Respir Med.

[7] Ranganath N.K., Malas J., Phillips K.G. (2020). Single and double lung transplantation have equivalent survival for idiopathic pulmonary fibrosis. Ann Thorac Surg.

[8] Smits J.M., Melman S., Mertens B.J. (2003). The Eurotransplant Study on Twin Lung Transplants (ESOTWIN): 90 paired single-lung transplants from the same donor. Transplantation.

[9] Jawitz O.K., Raman V., Becerra D., Klapper J., Hartwig M.G. (2022). Factors associated with short- versus long-term survival after lung transplant. J Thorac Cardiovasc Surg.

[10] Yusen R.D., Shearon T.H., Qian Y. (2010). Lung transplantation in the United States, 1999-2008. Am J Transplant.

[11] Meyers B.F., Lynch J.P., Trulock E.P., Guthrie T., Cooper J.D., Patterson G.A. (2000). Single versus bilateral lung transplantation for idiopathic pulmonary fibrosis: a ten-year institutional experience. J Thorac Cardiovasc Surg.

[12] Thabut G., Christie J.D., Ravaud P. (2009). Survival after bilateral versus single-lung transplantation for idiopathic pulmonary fibrosis. Ann Intern Med.

[13] Weiss E.S., Allen J.G., Merlo C.A., Conte J.V., Shah A.S. (2009). Survival after single versus bilateral lung transplantation for high-risk patients with pulmonary fibrosis. Ann Thorac Surg.

[14] Speicher P.J., Ganapathi A.M., Englum B.R. (2015). Single-lung transplantation in the United States: what happens to the other lung?. J Heart Lung Transplant.

[15] Schaffer J.M., Singh S.K., Reitz B.A., Zamanian R.T., Mallidi H.R. (2015). Single- vs double-lung transplantation in patients with chronic obstructive pulmonary disease and idiopathic pulmonary fibrosis since the implementation of lung allocation based on medical need. JAMA.

[16] Chauhan D., Karanam A.B., Merlo A. (2016). Post-transplant survival in idiopathic pulmonary fibrosis patients concurrently listed for single and double lung transplantation. J Heart Lung Transplant.

[17] Li D., Liu Y., Wang B. (2020). Single versus bilateral lung transplantation in idiopathic pulmonary fibrosis: a systematic review and meta-analysis. PLoS One.

[18] Gerbase M.W., Spiliopoulos A., Rochat T., Archinard M., Nicod L.P. (2005). Health-related quality of life following single or bilateral lung transplantation: a 7-year comparison to functional outcome. Chest.

[19] Mason D.P., Rajeswaran J., Murthy S.C. (2008). Spirometry after transplantation: how much better are two lungs than one?. Ann Thorac Surg.

[20] Tsagkaropoulos S., Belmans A., Verleden G.M. (2011). Single-lung transplantation: does side matter?. Eur J Cardiothorac Surg.

[21] Snell G.I., Shiraishi T., Griffiths A. (2000). Outcomes from paired single-lung transplants from the same donor. J Heart Lung Transplant.

[22] Langer F., Lepper P.M., Weingard B., Aliyev P., Bals R., Wilkens H. (2024). Two single lung transplantations from one donor: lung twinning in the LAS era. Respir Res.

[23] Hussain M., Thornton M., Hussain T. (2023). Evaluating the use of CT-derived lung volumes in donor-recipient lung size matching for lung transplantation in patients with interstitial lung disease and/or idiopathic pulmonary fibrosis. Transplant Proc.

[24] Miyoshi S., Schaefers H.J., Trulock E.P. (1990). Donor selection for single and double lung transplantation. Chest size matching and other factors influencing posttransplantation vital capacity. Chest.

[25] Miyoshi S., Demertzis S., Eckstein F., Hohlfeld J., Schaefers H.J. (1999). Chest size matching in single and double lung transplantation. Jpn J Thorac Cardiovasc Surg.

[26] Ramos K.J., Harhay M.O., Mulligan M.S. (2019). Which shall I choose? Lung transplantation listing preference for individuals with interstitial lung disease and chronic obstructive pulmonary disease. Ann Am Thorac Soc.

